# HIV Protein Sequence Hotspots for Crosstalk with Host Hub Proteins

**DOI:** 10.1371/journal.pone.0023293

**Published:** 2011-08-15

**Authors:** Mahdi Sarmady, William Dampier, Aydin Tozeren

**Affiliations:** Center for Integrated Bioinformatics, Drexel University, Philadelphia, Pennsylvania, United States of America; Inserm U869, France

## Abstract

HIV proteins target host hub proteins for transient binding interactions. The presence of viral proteins in the infected cell results in out-competition of host proteins in their interaction with hub proteins, drastically affecting cell physiology. Functional genomics and interactome datasets can be used to quantify the sequence hotspots on the HIV proteome mediating interactions with host hub proteins. In this study, we used the HIV and human interactome databases to identify HIV targeted host hub proteins and their host binding partners (H2). We developed a high throughput computational procedure utilizing motif discovery algorithms on sets of protein sequences, including sequences of HIV and H2 proteins. We identified as HIV sequence hotspots those linear motifs that are highly conserved on HIV sequences and at the same time have a statistically enriched presence on the sequences of H2 proteins. The HIV protein motifs discovered in this study are expressed by subsets of H2 host proteins potentially outcompeted by HIV proteins. A large subset of these motifs is involved in cleavage, nuclear localization, phosphorylation, and transcription factor binding events. Many such motifs are clustered on an HIV sequence in the form of hotspots. The sequential positions of these hotspots are consistent with the curated literature on phenotype altering residue mutations, as well as with existing binding site data. The hotspot map produced in this study is the first global portrayal of HIV motifs involved in altering the host protein network at highly connected hub nodes.

## Introduction

Hub proteins in the human protein network undergo transient binding interactions with hundreds of interaction partners, as quantified in the Human Protein Reference Database (HPRD) [1]. Using protein-protein interaction data involving pathogen strains, Dyer et al. [2] illustrated the tendency of pathogen proteins to preferentially interact with host hub proteins. Recent bioinformatics studies also demonstrated a significantly greater propensity for HIV to interact with highly connected host proteins [3], [4]. Multiple and repeated domains were shown to be enriched in date hub proteins along with long disordered regions [5], suggesting a mechanism for their ability to undergo transient interactions. Pairs of strings of domains are highly predictive of hub protein binding to other host proteins in phosphorylation events [6], however, domain-motif interactions appear to dominate phosphorylation of HIV proteins by host kinases [7].

The HIV-1, Human Protein Interaction Database (HHPID) [8] identifies about twenty host hub proteins with at least one hundred binding partners as directly binding to one or more HIV proteins. Some of these hub proteins phosphorylate their partners, while others cleave or recognize HIV protein sequences for nuclear localization. The high copy number of viral proteins in infected cells may lead to the out-competition of host proteins for their interaction with hub proteins as part of the topology of signaling and metabolic protein networks [9]. To quantify the changes imposed on the host protein network by HIV, it would be important to identify the hotspots on HIV protein sequences that are used to interact with hub proteins. Such hot spots could represent potential antiretroviral drug targets [10], [11], [12]. Moreover, sequence patterns of such spots could be used to identify host proteins outcompeted by viral proteins using the concept of motif sharing for hijacking a host protein function [3], [13]. Viral proteins can mimic native interfaces and thus interfere with binding events in host protein networks [14].

In this study, we used the identity of HIV targeted host hub proteins as input, along with sequences of their binding partners and the multiple alignments of HIV proteins, in order to identify hotspots along the viral protein sequences for binding to host hubs. Motivation for this study comes from recent system-wide studies highlighting the importance of HIV targeted host hub proteins in the course of infection [15], [16], [17]. The approach used in the present analysis for identifying sequence hotspots is based on motif discovery and motif enrichment statistics. It is well established that linear sequence motifs, 3 to 10 amino acids long, play important roles in transient binding interactions among proteins [18], [19]. However, eukaryotic linear motifs documented in the literature appear to be too general and ubiquitous to be discriminating between false positives and false negatives [4], [7], [9].

Our high throughput approach to motif discovery is specific to motifs shared by pathogen and host proteins. In this particular case, we set out to discover short linear protein motifs, which are (a) highly statistically enriched among neighbors of host hub proteins and (b) highly conserved in the varying sequences of HIV proteins. If a motif is highly conserved on known sequences of at least one HIV protein, it is likely that the motif is essential to viral infectivity. Secondly, an HIV motif involved in binding to a hub protein is likely to be present on the sequences of host proteins competing with HIV for transient binding interactions with the hub protein. In our previous work, we showed that this was the case for eukaryotic linear motifs [9].

Multiple methods and approaches have been developed for *de novo* motif discovery using protein sets and protein interactome datasets [14], [20], [21], [22], [23], [24]. Discovery of correlated motifs on binding partners in an interactome subset reduces the discovery of motifs with no apparent function [24], but is not readily suitable to the present case of identifying motifs on large numbers of proteins interacting with the same hub. As in the correlated motif discovery approach, our method utilizes protein-protein interactions, but the dataset we use for motif discovery is highly asymmetric containing only nineteen hub proteins on one side and their more than a thousand binding partners on the other side. We employed the SLiMFinder tool [21] for *de novo* motif discovery in this context, as it is comprehensive, customizable and has extensive documentation. For each HIV targeted hub protein, we identified the set of host proteins that interact with the hub protein using HPRD and added to this list multiple sequences of HIV proteins known to bind to the hub protein. We created such sequence sets containing hundreds of protein sequences for motif discovery. The resulting lists of motifs were further tested for their statistically enriched presence among hub neighbors in comparison to the HPRD proteins. Motifs that passed the test were further considered for their conserved expressions on hundreds of multiple alignments of HIV proteins known to interact with hub proteins. Our approach identified discrete sets of hotspots on HIV protein sequences potentially involved in HIV - host hub interactions. Our method recaptured the identities of eukaryotic linear motifs known to interact with host hub proteins. An extensive literature search showed functional validity of a dozen hotspots with previously unknown motifs, indicating the biological importance of the motif discovery presented in this study.

## Results

In this study, we set out to discover linear protein sequence motifs shared by HIV protein sequences and a large subset of the immediate neighbors of host hub proteins targeted by HIV. We combined randomly chosen viral protein sequences with the sequences of proteins known to interact with HIV targeted hub proteins to generate motif discovery sequence sets, one hub protein at a time. We used the SLiMFinder motif discovery algorithm to identify motifs that are not only conserved on HIV sequences but also statistically enriched among neighbors of HIV targeted hub proteins. Table 1 lists the gene IDs and gene symbols of these hub proteins, along with the number of binding partners and the GO molecular functions of these neighbors. Also shown in the table are the identities of HIV proteins interacting with these hub proteins. HIV Tat and Nef interact with 9 and Gag with 7 of the hub proteins listed in Table 1. HIV targeted hub proteins considered in this study comprise mostly of kinases and transcription factors. Some of the hub proteins listed in Table 1 exist in complexes in vivo. Transient binding of an HIV protein to such a complex may involve binding interactions with multiple host proteins. For example, experimental evidence pointing to viral proteins binding to p53 associated with CREBBP/EP300 activators, forming transient ternary complexes [25]. In this study, we consider, these three host proteins as if each interacting physically with an HIV protein (Table 1). Such an approach is based on the concept of outcompetition of host proteins by viral proteins and will yield false positives if HIV proteins bind to the other proteins in the complex and not the hub protein under consideration. However, all hub proteins listed in Table 1 have been deemed as directly binding to at least on HIV protein in research literature.

**Table 1:** List of host hub proteins targeted by HIV.

Entrez ID	Symbol	Neighbors Count	GO Molecular Function	HIV-1 Protein Interactor
7157	TP53	266	TF, RNA binding, DNA binding	Nef, Tat
2033	EP300	210	TF activator	Tat, Vpr
6714	SRC	208	kinase, RNA binding	Nef
1387	CREBBP	198	TF activator	Tat, Vpr
5578	PRKCA	173	kinase, RNA binding	Gag, Nef, Pol, Rev, Tat
1457	SNK2A1	169	kinase, RNA binding	Gag, Pol, Rev, Vpu
5594	MAPK1	160	kinase, kinase binding, RNA binding	Gag, Nef, Rev, Tat, Vif
2534	FYN	154	kinase, RNA binding	Nef
5566	PRKACA	145	kinase, kinase binding, RNA binding	Gag, Nef
5295	PIK3R1	128	protein phosphatase binding	Nef
983	CDC2	119	kinase, RNA binding	Rev
5595	MAPK3	116	kinase, RNA binding	Tat, Vif, Gag, Rev
3725	JUN	116	TF, DNA binding	Tat
801	CALM1	114	phosphorylase kinase	ENV, Gag, Nef, Pol
7431	VIM	112	kinase binding	Pol
5970	RELA	111	kinase binding, TF	Tat
3932	LCK	105	kinase, kinase binding, RNA binding	Nef
5580	PRKCD	102	kinase, RNA binding	Pol, Tat
60	ACTB	101	kinase binding, RNA binding	Gag, Pol

The table lists HIV targeted human proteins with more than 100 immediate neighbors in HPRD. Also listed are the numbers of neighbors of hub proteins and GO Molecular Functions enriched among neighbors against the background set of HPRD proteins. The last column identifies the HIV proteins targeting the hub proteins.

### HIV protein sequence hotspots for binding to host hubs

**Figure 1:**
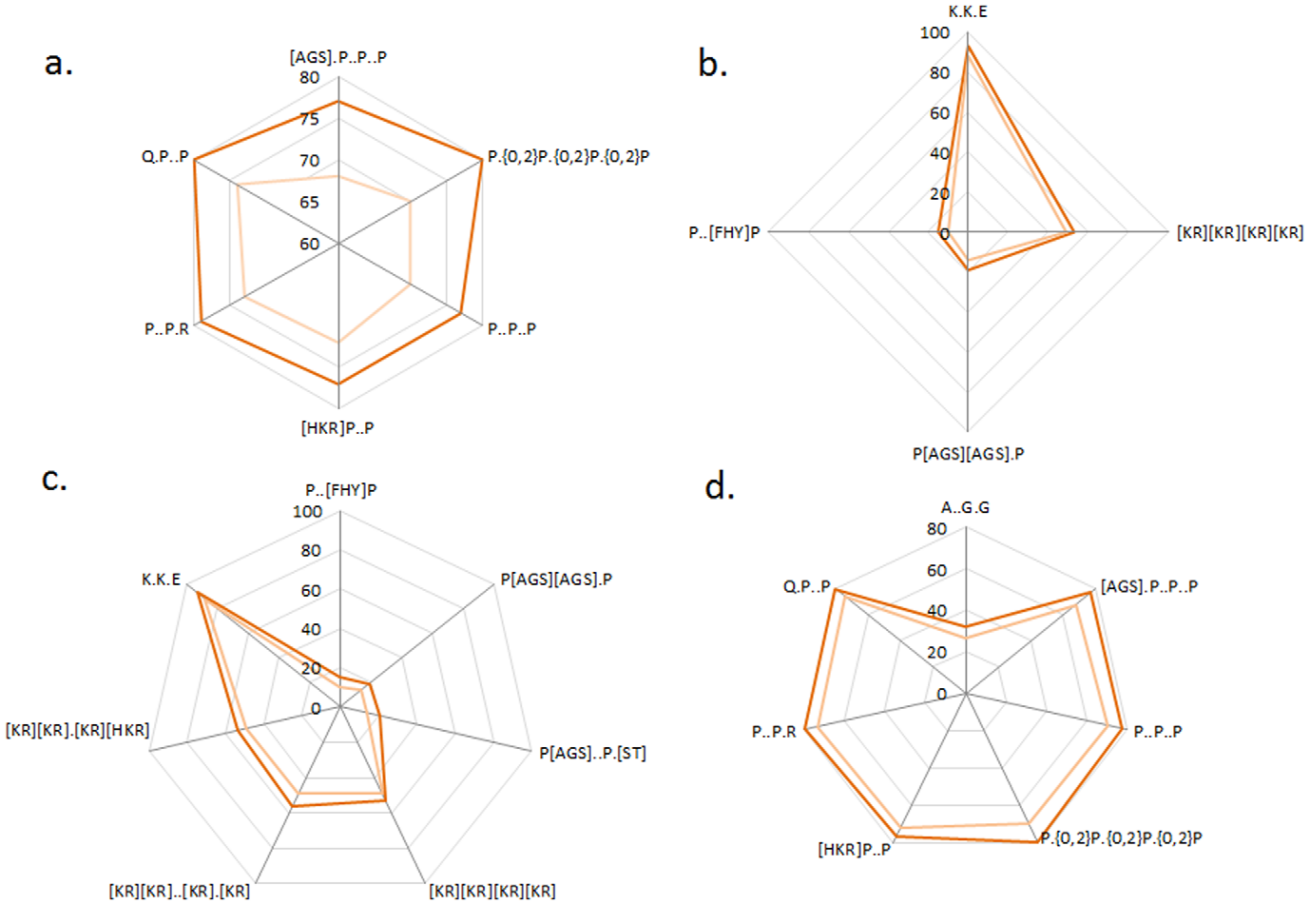
Radar graphs visualizing predicted motifs positions on NEF and TAT. The radar graph illustrating the computationally predicted motifs on Nef for binding to SRC (1a, 1c) and Tat for binding to EP300 (1b, 1d) at p value cut offs of 0.005 for 1a and 1b and 0.01 for 1c and 1d. The radial distance indicates amino acid residue number on the HIV protein sequence starting from the N terminal. Edges of hotspots are marked with orange lines.

Our computations indicate HIV protein sequence motifs involved in binding interactions with host hub proteins concentrate on distinct spots on the sequence. Shown in Figure 1 is a typical result of motif discovery, presented for the sets of motifs potentially involved in binding to host hub proteins, with their positions specified on HIV proteins. The radar plots in Figure 1 illustrate computationally predicted motifs on Nef for binding to SRC (1a, 1c) and Tat for binding to EP300 (1b, 1d) at p value cut offs of 0.005 for 1a and 1b and 0.01 for 1c and 1d. The p value in this figure reflects the statistical enrichment of the discovered motifs on the binding partners of host hub proteins SRC and EP300 with respect to their expression among host proteins listed in HPRD. The figure shows that the number of discovered motifs decreases with increasing statistical significance. More detail on each motif shown in the figure is available in Table S1. The radar plot organizes discovered motifs on circles with a radius equal to the sequence distance from the start of the protein sequence to the start of the discovered motif. The figure shows that predicted motifs rich in proline and related to the LIG_SH3 ELM pattern are spatially clustered along the sequence of HIV Nef. Consolidation of these motifs into one pattern is possible with the use of a regular expression; however, the motifs shown may have slightly different functions, similar to the multiple ELMs known to interact with SH1, SH2, and SH3 protein domains.

**Figure 2:**
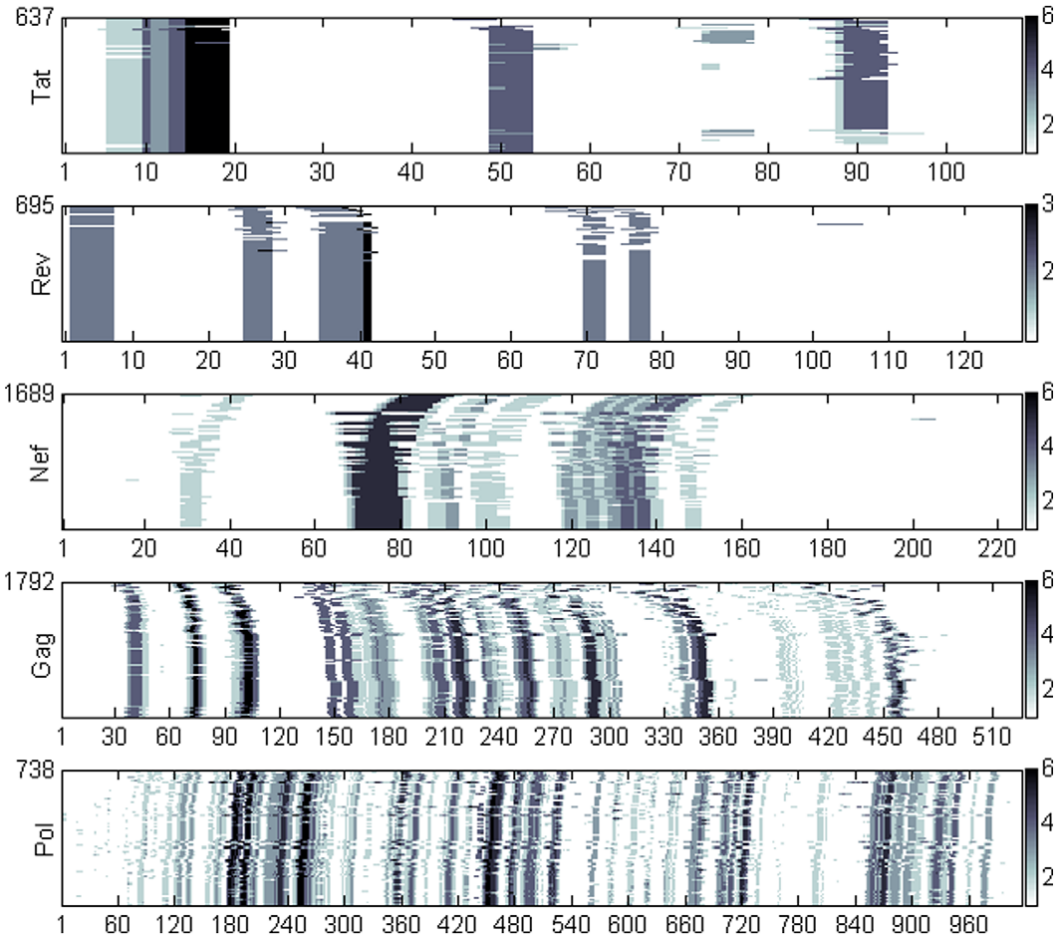
Hotspots on HIV protein sequences. Amino acid sequence positions of motif hotspots are shown on the horizontal axis. The vertical axis identifies the number of viral protein sequences in the alignment. Color intensity is proportional to the number of hub proteins with enriched hotspot motifs among its immediate neighbors. Regions highlighted in this figure have at least two different hub proteins associated with them.

The motifs discovered on the binding partners of multiple hub proteins fall onto the same hotspot on HIV proteins. The sequence hotspots for HIV proteins Tat, Rev, Nef, Gag, and Pol are shown in Figure 2, where the motifs are projected onto multiple alignments of HIV proteins, ranging from 637 sequences for Tat to 1792 sequences for Gag. The amino acids along the HIV protein sequence are painted with gray scale intensities proportional to the number of hubs associated with a motif on that sequence position. The figure shows increasing entropy on hotspot positions with increasing sequence length and sequence copy number. Aligning sequences for optimizing positional conservation requires too many gap insertions and thereby distorts the actual positions of these motifs and thus we avoided this route. The figure shows four hotspots on Tat, five on Rev, eight on Nef, and significantly more on Gag and Pol. In our analysis, these hotspots comprise multiple sites for transient interactions with hub proteins.

**Figure 3:**
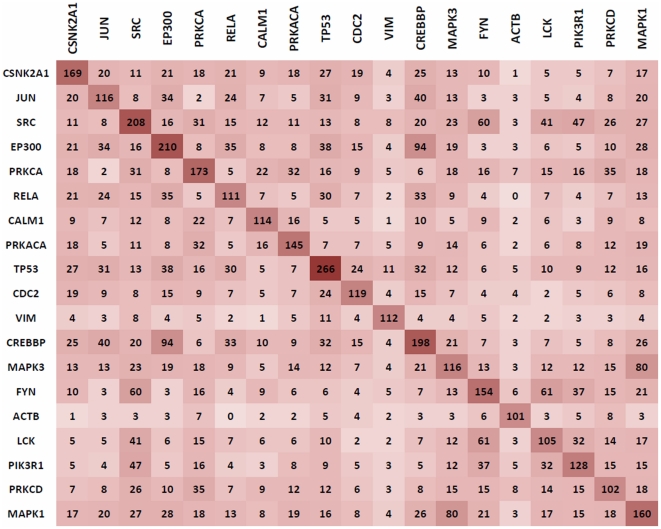
Heat map for common neighbors among hub proteins considered in the study. The number of common immediate neighbors between two hub proteins is show elements of a square matrix. Color intensity is proportional to the number of protein neighbors common to two hub proteins.

Next, we considered whether the hotspots shown in Figure 2 were mainly due to host hub proteins having large numbers of commonly shared binding partners. Our motif discovery approach depends on sequences of binding partners of host proteins. If two host hub proteins interacting with the same HIV protein have a large number of common binding partners, similar motifs discovered in the two motif discovery sets (one for each hub) would likely fall on to the same hotspot. Motifs presented in this study, found via SlimFinder motif discovery tool, are expressed in at least 20 percent of the binding partners of an HIV interacting host hub, a cut off chosen to focus on most dominant motifs. The heat map in Figure 3 showing numbers of common neighbors for pairs of HIV targeted hubs indicates large intersection (94 common binding partners) for binding partners of host hubs EP300 (with 210 partners) and CREBBP (with 198 partners). Similar large intersections exist for binding partners of MAPK1 and MAPK3; and FYN and SRC. The hotspot shown in between positions 2 to 7 on Rev in Figure 2 is indeed due to MAPK1 and MAPK3 having common neighbors. Thus, in some cases, viral protein hotspots may largely be made of motifs present on the common binding partners among HIV-protein interacting host proteins.

### Biological context for sequence hotspots

A subset of the HIV protein binding motifs discovered in this study corresponds to host linear motifs already annotated by the ELM web server. Shown in Table 2 are the ELMs that satisfy the three conditions we imposed on motif discovery, namely, these ELMs are (1) conserved along the HIV protein sequence, (2) expressed infrequently on HPRD proteins, and (3) statistically enriched among the neighbors of hub proteins. The start and end positions of ELMs on HIV protein sequences are indicated in the table. Any ELM motif satisfying these conditions was included in the table, regardless of whether they were deemed functional or not in an experimental study. Some of the motifs in the Table (those annotated with PUBMED references) have already been associated with the specific virus-host protein binding events cited in the table. The ELM motif LIG_SH3-2, a kinase associated motif, is present on HIV proteins Env, Gag, and Nef. It was previously implicated in binding of Env to CALM1 [26]. The nuclear localization signal motif TRG_NLS_MonoCore_2 is found on Tat and Pol and was implicated in interactions with CSNK2A1 [27]. The PCSK cleavage site is conserved on Rev and Pol and shown to be involved in binding interactions with CALM1 [28], [29]. The immune-receptor tyrosine-based switch motif is found expressed on Env and has been previously linked to HIV [30]. The SH3-2 motif in Table 2 was also listed in Table 1 of a recent review article on how viruses hijack cell regulation [31] as an example of viral mimicry of host motifs. The other motifs found in our Table but absent in Davey et al. [31] such as CLV_PCSK-PC7_1 will have to be annotated experimentally for the binding event functions listed in our table. The fact that our method reproduced all of the eukaryotic motifs on HIV proteins satisfying our stringent criteria attests to the effectiveness of the motif discovery approach used in the study.

**Table 2:** Eukaryotic linear motifs (ELMs) present on HIV and enriched among neighbors of hub proteins.

HIV	ELM	Site	Hub	p-value	PUBMED Id
**Env**	MOD_TYR_ITSM	35–43	CALM1	9.80E-04	15731257
	LIG_SH3_4	117–125	CALM1	5.30E-04	PMC364842
**Gag**	LIG_SH3_2	288–294	CSNK2A1	2.79E-03	
			PRKCA	1.73E-03	
			MAPK1	1.92E-04	
			MAPK3	4.33E-04	
	LIG_SH3_1	451–458	MAPK1	4.24E-04	
**Nef**	LIG_SH3_2	72–78	FYN	2.67E-06	7859737
			PIK3R1	6.08E-06	
			PRKCA	1.73E-03	
			MAPK1	1.92E-04	8794306
			SRC	2.18E-11	16849330
**Pol**	CLV_PCSK_PC7_1	233–240	CALM1	7.79E-04	20121080
	TRG_NLS_MonoCore_2	255–261	CSNK2A1	2.99E-03	18818209
**Rev**	CLV_PCSK_FUR_1	39–44	CSNK2A1	4.01E-03	
**Tat**	TRG_NLS_MonoCore_2	48–54	EP300	2.99E-05	11080476
			JUN	1.28E-03	
			RELA	1.18E-03	
			TP53	5.78E-04	
			CREBBP	1.88E-05	
**Vif**	LIG_SH3_1	158–165	MAPK1	4.24E-04	9792705

The *sequence sites* of ELMs are specified based on the most commonly observed start and end points on the HIV protein sequences. The *p-values* listed stand for the statistical enrichment of the ELM among neighbors of the hub protein using hypergeometric test. The PUBMED IDs indicate the research articles identifying the binding site experimentally.

A semi-automated literature search on directed mutagenesis of HIV sequences came up with 24 research articles presenting HIV mutations intersecting with motifs predicted in this study. Fourteen of these mutations corresponded to known phenotype changes in HIV-host interactions (Table 3). The hotspot positioned at residues 15–19 of Tat contained mutation S16A that is known to prevent Tat phosphorylation. The hub protein interacting with Tat at this position is PRKCD, a kinase known to phosphorylate Tat. The second set of mutations (R52Q, R53Q) fell onto the hotspot intersecting with TRG_NLS_MonoCore ELM, a motif recognized by the importer protein importin-alpha. Some of the motifs expressed by Vif, Vpr, and Vpu (presented in Table S1) intersected with mutations known to affect viral protein activity (Table 3).

**Table 3:** Directed mutations of HIV protein sequence in research literature within the range of motifs annotated in this study.

HIV-1	Pubmed ID	Mutation	Motif Pattern	Hub Symbol	Start	End	Mutation Phenotype
**Gag**	9420228 [45]	P222A	A.{0,1}G.{0,2}P.{1,2}P	CSNK2A1	217	223	Diminishes virion incorporation of CyPA and interfere with HIV-1 replication
			P..PG	MAPK3	219	224	
**Nef**	10547288 [46]	F90R	[DE]L..[FIL][IL]	MAPK1	87	93	Reduces the affinity of SH3 binding (specifically in HCK, similarly in FYN, LCK)
			[DE]L..[FL]L	LCK	87	93	
			F.{0,2}L.{0,2}K	FYN	91	94	
	10489340 [47]	R77A	P..P.R	PIK3R1, SRC, MAPK1	73	79	Decreases downregulation of class I MHC
**Pol**	20450778 [48]	K101P	K.K.I	PRKCD	98	103	Correlated with drug resistance (failing combinational antiretroviral therapy)
		K219	A..KK	ACTB	216	221	
		K70R	[KR][ILV]..Q.[KR]	CALM1	69	76	
**Tat**	8709193 [49]	R52Q, R53Q	KR..R	RELA	51	56	Repeals Tat binding to TAR element and gene trasactivation
			[AS]..R.[KR][KR]	JUN	46	53	
			[KR][KR][KR][KR]	EP300, CREBBP	49	53	
	17083724 [50]	S16A	[FHY]..[ST].P	PRKCD	13	19	Prevents Tat phosphorylation and interferes with activation of HIV-1 provirus
**Vif**	8626571 [51]	S144A	S.Q.L	MAPK3	144	149	Loss of Vif activity (not phosphorylated)
**Vpr**	14506268 [52]	I61A,L64P	E.[IL].[KR].[LV]	EP300	58	65	Enhances pro-apoptotic activity of Vpr
	9557700 [53]	Q65E	QQL	CREBBP	65	68	Impairs Vpr nuclear localization
**Vpu**	20078884 [54]	S52A	[AGS]..S..E.[DE]	CSNK2A1	50	59	Interrupts phosphorylation of Vpu required for degradation of tetherin

The table lists the HIV protein, the PUBMED ID presenting the directed mutation, motif pattern, hub symbol, the start and end of the motif and the mutation phenotype.

A subset of our predicted HIV protein regions binding to hubs in Table 1 was previously identified in the literature. Shown in Table 4 are sixteen experimentally annotated binding sites, ten of which (shown in italics) match our binding predictions both in terms of sequence position of the binding site as well as the targeted host hub. In all these cases, predicted sequence position is within the experimentally annotated position. The table also lists 5 cases where experiments and predictions are not identical but related. Experimentally annotated binding sites to CREBBP and EP300 appear interchanged in our prediction set. These proteins are often associated with each other and have common binding partners. In another instance, we predict Rev binding site to CSNK2A1 to be at the edge of the experimental binding site. Discrepancy could be due to variation of the length of the Tat sequence used in experimental annotation from the most frequently found length in our Tat sequence collection used in motif discovery. We also found predicted VIM, MAPK1, MAPK2, VIM binding sites on Env matching experimentally annotated Env binding site to CALM1. Overall, the Table shows the promise of our approach to critically examine the experimental results available in the literature on host target binding sites on HIV proteins.

**Table 4:** Experimentally determined binding sites of HIV-1 proteins to hub proteins and their intersection with motifs discovered in our analysis.

HIV	HUB	PUBMED ID	Literature Binding Site	Predicted binding site	Predicted Hub	Motif
**Env**	PKKCA	1832084 [55]	581–597	588–594	PKKCA	[FWY].[KR]
**Env**	CALM1	8226798 [56]	768–788	782–788	VIM, MAPK1	[HKR]..{E[IL]
**Env**	CALM1	8226798 [56]	826–854	843–848	CDC2	P.R.R
**Gag**	CALM1	11054265 [57]	11–46	38–55	CALM1	S.E..R
**Nef**	CALM1	15632291 [58]	1–20	16–22	CALM1	[FILV][KR][DE]..[HKR]
**Nef**	MAPK1	8794306 [59]	69–78	70–77	MAPK1	P..P..P
**Nef**	LCK	8794306 [59]	69–78	70–80	LCK	P..P..P
**Nef**	FYN	7859737 [60]	65–82	73–78	FYN	EE..S
**Tat**	CREBBP	12549909 [61]	1–24	10–15	EP300	P..[FHY]P
**Tat**	EP300	11080476 [62]	48–57	49–56	CREBBP	[KR}[KR}..[KR].[KR]
**Vif**	MAPK1	9792705 [63]	96–165	161–165	MAPK1	PP.P
**Vpr**	EP300	12208951 [64]	64–84	58–65	EP300	E.[IL].[KR].{LV]
**Vpu**	CSNK2A1	8548340 [65]	52–56	52–59	CSNK2A1	[DE].S..[DE].[DE]
**Rev**	CSNK2A1	8806671 [66]	5–8	None	None	
**Rev**	CSNK2A1	11827166 [67]	33–50	41–49	CSNK2A1	[KR]N.{HKR]
**Rev**	CSNK2A1	11827166 [67]	12–24	24–28	CSNK2A1	Q [AGS].P

The last column indicates typical regular expressions of motifs in that hotspot.

**Figure 4:**
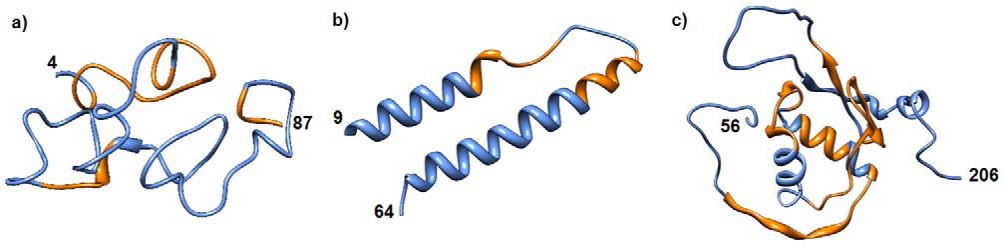
Hotspots on HIV protein structures. Hotspot regions highlighted in orange on Tat (a), Rev (b), and Nef (c) proteins. PDB structures 1TBC [41], 2X7L [42], and 2NEF [43] were used respectively. Numbers on the structures reflect the start and stop positions on the actual HIV protein sequence. Molecular graphics images were produced using the UCSF Chimera package [44].

Next, we mapped our predicted hotspots to the 3D structures of three of the smaller HIV proteins. The structures for Tat, Rev, and Nef were retrieved from the protein data bank (PDB) [32], and hotspots on these structures were highlighted in orange in Figure 4. The figure clearly shows that the hotspots we identified do not form conformational recognition features. More likely, these hotspots are being utilized in anchoring two proteins at multiple sites. Redundancy of binding motifs on HIV Nef for the same host protein was recently illustrated [33].

Our results point to predicted motifs rarely containing amino acid residues often found buried in 3D structure of a protein. We have tested solvent accessibility of the motifs on the hotspots shown in Figure 4. The hotspots on Tat, Rev, and Nef in this figure correspond to Tat hotspots 10–19, 48–54, and 87–94; Rev hotspots 24–28 and 34–43; and Nef hotspots 28–32, 68–80, and 120–138 in Figure 2. We have identified the discovered motifs in these hotspots and computed the fraction of surface accession of the motifs in these hotspots along HIV proteins. Briefly, we assumed amino acid residues R, K, E, D, Q, and N as highly solvent accessible residues and used the symbol s to be the fraction of occurrence of these hydrophilic residues on the motif representing the hotspot [34]. We determined similar ratios (n, b) for neutral residues P, H, Y, G, A, S, and T; and for hydrophobic residues C, V, L, I, M, F, and W. Results of these computations for hotspots in Figure 4 are presented in Table 5. It is clear from this table that motifs contained in the hotspots are mostly composed of hydrophilic and neutral residues, indicating solvent access.

**Table 5:** Surface accessibility composition (hydrophilic, neutral, and hydrophobic) of motifs in HIV protein hotspots along the collections of viral protein sequences.

HIV Hotspot	Motif	Regular Expression	s	n	r
Tat 10–19	14–19	P[AGS][AGS].P	0.20	0.80	0.0
Tat 49–54	49–54	[KR].[KR][KR][KR]	1.0	0.0	0.0
Tat 88–93	88–93	K.K.E	0.80	0.20	0.0
Rev 24–28	24–28	Q[AGS].P	0.50	0.50	0.0
Rev 34–43	38–43	[KR][KR].[KR][HKR]	1.0	0.0	0.0
Nef 29–33	29–33	G.GA	0.0	0.75	0.25
Nef 68–80	70–80	P.{0,2}P.{0,2}P.{0,2}P	0.29	0.41	0.29
Nef 120–138	130–136	P.P..[HKR]	0.17	0.71	0.12

First column depicts the start -end positions of hotspots on viral proteins shown in Figure 4. The second and third columns represent start-end positions of motifs in these hotspots and their regular expression. The last three columns depict the fractions of hydrophilic (s), neutral (n) and hydrophobic (b) residues in these motifs. Only one motif per hotspot is shown.

## Discussion

HIV alters the host cell macromolecule network and redirects cellular processes towards the synthesis of new viral particles. Binding interactions of HIV proteins with host proteins, DNA, and RNA constitute a fundamental mechanism in the modification of host cellular networks in favor of synthesis of viral particles. Network connectivity is significantly affected by the binding of viral proteins to host hub proteins. As shown in Table 1, nineteen such host proteins with at least 100 binding partners appear as directly interacting with HIV proteins in HHPID. HIV-targeted host hub proteins are typically protein kinases and/or transcription factors. Therefore, alterations in their connectivity directly impacts signal flow through pathways and potentially leads to significant changes in global gene expression profiles.

Given that an HIV protein binds to a host hub protein, what can we say about the altered connectivity of the hub protein? One scenario would be that binding of the HIV protein to the hub protein occurs at sites utilized by host proteins to bind to the hub. Examples of such sites include phosphorylation and docking sites [7]. Even if phosphorylation of an HIV protein turns out to have little functional consequence on its own, the fact that multiple host proteins are outcompeted by the thousands of copies of the HIV protein would implicate a strong impact on network connectivity on the hub node under consideration. This is the rationale for the focus of the present study on the grammar of interactions between HIV and host hub proteins.

This study presents sets of newly annotated hotspots on HIV virus proteins as potential sites for binding to host hub proteins. The hotspots are at the intersections of short linear motifs shared by HIV proteins and the host proteins outcompeted by HIV proteins. We used a de novo motif discovery algorithm [21] with sequence data as the input, consisting of HIV and host protein sequences, as described in the methods. The output consisted of motifs shared by the HIV and the host proteins competing in binding events to host hub proteins. The motifs discovered in this study are (i) conserved on HIV protein sequences, (ii) found in less than one-third of the host proteins, and (iii) are statistically enriched among neighbors of host hubs targeted by HIV proteins. The sequence positions of these motifs on the HIV proteins constitute potential binding sites for host hubs. Thus, through a convoluted bioinformatics approach requiring extensive data on protein sequences and interactomes, we predict the interface between HIV and host hub proteins.

Our computational estimates of hotspots along the sequence of HIV proteins identified already known eukaryotic linear motifs associated with nuclear localization signal on Tat and Pol, a PCSK mediated cleavage site on Rev and Pol, and a proline-rich kinase substrate motif on Env, Gag, and Nef (Table 2). In fact, our method reproduced all the eukaryotic linear motifs satisfying the stringent criteria we imposed on their expression on HIV and on the neighbors of hub proteins. Our findings are also in line with large-scale experimental data on directed mutagenesis of the HIV sequences. Fourteen phenotype-altering single residue changes of HIV proteins collected from the literature were mapped onto the hotspot locations (Table 3). Additionally, our predictions recaptured a large majority of the known interfaces between HIV and hub proteins (Table 4). To our knowledge, the large-scale motif analysis presented in this study constitutes the first comprehensive map predictive of HIV-host hub binding interfaces. It was possible to create a hotspot map for the HIV proteome thanks to the extensive research findings in the literature on the identity of host hub proteins interacting with HIV proteins.

The predicted results for HIV motifs presented in this study do not recapture all known HIV protein linear motifs involved in communication with the host. In a number of cases, the motif discovery tool correctly identifies the motifs as output but we eliminated such motifs due to statistical constraints we imposed involving their presence among hub neighbors. An example for this case is the RKGLGI motif, conserved between HIV-1, HIV-2 and SIV Tat [35]. Similarly, our approach eliminates those motifs not found on majority of HIV protein sequences of certain type and thus might be missing important motifs linked to infectivity. Such motifs can always be recaptured for further study about their involvement in the course of HIV infection.

Potential uses of HIV sequence hotspots depicted in this study range from drug development to better understanding of the mutation phenotypes in their linkage to host protein networks. Rational drug design procedures are increasingly focusing on developing drugs targeting protein-protein interaction interfaces [10]. The data produced by our study shows that the specific motif sequence segments expressed by viral proteins are often different than the motif sequences commonly used by the host. This provides an opportunity to block the binding interactions of HIV proteins with host hubs using peptides or small molecules, without affecting hub connectivity to other host proteins. Another potential use is to provide biological context for mutation phenotypes that may be expressed in general terms, such as loss of viral infectivity [36].

Hotspots produced by our method linked phenotype altering mutations on HIV proteins to the identity of the host protein it interacts with at the site of mutation, allowing the use of bioinformatics in outlining a protein network pathway responsible for the phenotype. The motif collection presented in Table S1 is a comprehensive list of protein motifs shared by host hub neighbors potentially outcompeted by HIV. The size of the hub neighbor protein set expressing a given motif provides a first order approximation of the identity of hub neighbors potentially outcompeted by HIV. Recently obtained crystal structure of HIV-1 Tat complexed with human P-TEFb provides further evidence that viral and host proteins interact on multiple sites, even in such rapid interaction events as phosphorylation [37]. One could further refine the predicted outcompeted protein set by identifying those hub neighbor subsets enriched with an expression of multiple motifs positioned at different hotspots along the viral protein.

The motif sets presented in this study could be refined further by future bioinformatics studies utilizing structural information. Consideration of motifs within the context of a structural organization of proteins, such as their presence on helical loops [38] and disordered regions [39], may lead to a better understanding of the grammar of the HIV virus - host protein interactions and the role of short linear motifs in these interactions. Additionally, correlated motif approaches detailed in the literature [24] provide a map for identifying the interface on the hub protein interacting with a hotspot on the viral protein.

## Methods

### Data Acquisition

Human protein interaction data were downloaded from the Human Protein Reference Database (HPRD) [1], Release 8, and HIV, human protein interaction data were obtained from HHPID [8] [8] (accessed December 2009). Eukaryotic linear motif (ELM) patterns were collected from the ELM resource [40]. We used the HIV-1 Sequence Database (http://www.HIV-1.lanl.gov/) for subtypes A, B, C, and D (2008 version) to download multiple protein alignments of HIV proteins (Env, Gag, Nef, Pol, Rev, Tat, Vif, Vpr and Vpu).

### Dataset preparation and motif discovery

Among the human proteins annotated as directly interacting with at least one HIV protein in HHPID, nineteen had at least 100 immediate neighbors in the HPRD database. The choice of 100 as a lower bound for the number of neighbors of a hub protein is arbitrary to some extent, as some known human hub proteins such as CDK1 have a lower number of binding partners. Our preliminary studies showed that the automated approach we used for motif discovery required significant computing time with increasing numbers of sequence batches and increasing numbers of sequences and lengths of sequences in each batch. The choice was also guided by our preliminary computations indicating that no new hotspots were annotated on the HIV sequence as the number of hub proteins considered reached from seventeen to nineteen. Interaction modes of the host hub proteins with HIV proteins under consideration were described in HHPID as “binds,” “phosphorylates” or “cleaves.”

In motif discovery, we sought motifs satisfying the following conditions: (1) conserved on multiple alignments of HIV proteins and (2) over-represented among proteins that share a common function, i.e., interacting with the same hub protein. Thus, the sequence set for motif discovery associated with a specified hub protein and an HIV protein consisted of the sequences of all host proteins binding to the hub protein, as well as sequences of the HIV protein equal in number to the closest larger integer to ten percent of the number of hub neighbor sequences. The HIV protein sequences used in motif discovery were chosen randomly from the collection of sequences. In the case of the hub protein TP53 with 266 neighbors, 27 randomly chosen Nef sequences were added to the dataset. Repeated random selection of HIV sequences in this manner did not result in new motif discovery. In total, 42 datasets, pairing 19 hub proteins with multiple HIV proteins were created for motif discovery.

The sequence datasets were fed into the motif discovery tool, SLiMFinder [21], for discovery of motifs ranging from 3 to 10 amino acids in length. The Blast e-value used in this tool was set to 1e-28. Other parameters for motif discovery in SLiMFinder were set to the default values in the tool manual. Motifs computed as output were first matched to human proteins to eliminate abundant motifs. Motifs present in more than one third of HPRD proteins were filtered. Our previous study based on eukaryotic liner motif annotation showed that motifs that were ubiquitously present were poor predictors of HIV- host interactions [9].

### Statistical enrichment

Statistical enrichment of discovered motifs among immediate neighbors of hub proteins was computed by using the hypergeometric test against the background expression in HPRD. Any protein containing at least one copy of a motif was deemed as motif expressing. We chose a p-value cutoff of 0.005 to eliminate non-significant motifs. Another requirement for further annotation of the discovered motifs is their conserved presence on the HIV sequences. Motifs that were not present on at least 70 percent of all of the major subtypes of the corresponding HIV protein sequence were removed. Since our approach is based on over representation of a motif among neighbors of a hub protein, we kept only those motifs that were present on at least 20 percent of the neighbors of the hub protein under consideration. Therefore, the final list of motifs for each hub-HIV protein dataset contained motifs, which are over represented and enriched among the neighbors of the hub protein, not abundant in the human proteome, and present on a vast majority of the sequences of HIV proteins interacting with hub proteins.

### Experimental data for comparison with predicted HIV sequence hotspots

Discovered motifs that passed the processing described above were projected onto protein sequences. Multiple motifs intersected along the sequence. Amino acid sequences belonging to multiple motifs comprised a set of hotspots. The intensity of the hotspot was deemed proportional to the number of hub proteins with motifs intersecting with the hotspot, normalized with respect to the number of hubs known to interact with the protein under consideration. Next, we searched PUBMED abstracts for directed mutagenesis studies involving mutations falling within the range of our motifs and hotspots. We also identified eukaryotic linear motifs conserved on HIV and statistically enriched among the neighbors of the hub proteins with the same cutoffs used in the motif discovery. We used these datasets to provide a biological context to the predicted HIV sequence hotspots for binding to hub proteins.

## Supporting Information

Table S1 **List of motifs defining hotspots per HIV protein.** This file is a nine-tab Excel spread sheet containing motifs shared by HIV proteins and some of the neighbors of HIV protein targeted hub proteins. Each tab lists motifs for an HIV protein with its corresponding details. Headings Hub ID and Hub Symbol represent the Entrez ID and gene symbol of the hub protein to which the motif belongs. Pattern is the regular expression of the motif. Info Content is the information content of the motif pattern. The p value is computed by statistical enrichment of the motif among neighbors of the hub protein in comparison to HPRD proteins. The number of neighbors of a hub protein and the neighbors on which the motif is present are shown with the symbols # of H2s and H2s w/Motif, respectively. Start and End headings refer to the start and end positions of the motif on the corresponding HIV protein sequence, calculated based on the most common positions observed on the HIV protein sequences.
(XLSX)
